# Wide-range viscoelastic compression forces in microfluidics to probe cell-dependent nuclear structural and mechanobiological responses

**DOI:** 10.1098/rsif.2021.0880

**Published:** 2022-04-20

**Authors:** Maria Isabella Maremonti, Valeria Panzetta, David Dannhauser, Paolo Antonio Netti, Filippo Causa

**Affiliations:** ^1^ Interdisciplinary Research Centre on Biomaterials (CRIB) and Dipartimento di Ingegneria Chimica, dei Materiali e della Produzione Industriale, Università degli Studi di Napoli ‘Federico II’, Piazzale Tecchio 80, 80125 Naples, Italy; ^2^ Center for Advanced Biomaterials for Healthcare@CRIB, Istituto Italiano di Tecnologia, Largo Barsanti e Matteucci 53, 80125 Naples, Italy

**Keywords:** single-cell, microfluidic device, viscoelastic forces, nuclear deformation, nuclear envelope

## Abstract

The cell nucleus plays a critical role in mechanosensing and mechanotransduction processes, by adaptive changes of its envelope composition to external biophysical stimuli such as substrate rigidity and tensile forces. Current measurement approaches lack precise control in stress application on nuclei, thus significantly impairing a complete mechanobiological study of cells. Here, we present a contactless microfluidic approach capable to exert a wide range of viscoelastic compression forces (10–10^3^ µN)—as an alternative to adhesion-related techniques—to induce cell-specific mechano-structural and biomolecular changes. We succeed in monitoring substantial nuclear modifications in Lamin A/C expression and coverage, diffusion processes of probing molecules, YAP shuttling, chromatin re-organization and cGAS pathway activation. As a result, high compression forces lead to a nuclear reinforcement (e.g. up to +20% in Lamin A/C coverage) or deconstruction (e.g. down to −45% in Lamin A/C coverage with a 30% reduction of chromatin condensation state parameter) up to cell death. We demonstrate how wide-range compression on suspended cells can be used as a tool to investigate nuclear mechanobiology and to define specific nuclear signatures for cell mechanical phenotyping.

## 1. Introduction

Cells are continuously exposed to mechanical stimuli, but the resulting subcellular responses are not fully understood. In recent years, it has been widely demonstrated that cells are able to sense and respond to different mechanical cues coming from their surrounding environment (e.g. compressive and shear forces, stiffness and substrate strain energy) [1–3]. Indeed, the transduction of such mechanical into biochemical signals relies on the ability of cells to perform mechanosensing processes, even leading to possible changes in cellular phenotypes [3–5]. In particular, cell nucleus plays a central role in mechanosensing with related changes in nuclear envelope (NE) composition, mainly constituted by the Lamin A/C and the nuclear pore complexes (NPCs), both deputed to finely tune chromatin re-organization—in the sense of condensation—and to regulate the trafficking of different re-localizing molecules [6–12]. In detail, the Lamin A/C network provides structural support and governs nuclear deformability and fragility [13]. High Lamin A/C expression levels are associated with stiffer and more viscous nuclei, whereas a deficiency of Lamin A/C correlates to both more deformable and fragile nuclei, possibly leading to nuclear breakage and cell death [13–16]. In adhesive conditions, thanks to a well-structured cytoskeleton, Lamin A/C expression and conformation have been detected to be directly involved in mechanosensing and mechanotransduction processes. In fact, the activation of production and/or recycling processes of Lamin A/C resulted to be closely regulated by human tissue-mimicking substrate stiffnesses [17,18]. Similarly, the translocation of Yes-associated Protein (YAP) transcription factor into the nucleus represents a typical mechanosensing phenomenon, observed during cell adhesion or migration. When the cytoskeleton is assembled, a signalling cascade arises from the imposed mechanical stimulus such as a variable substrate stiffness [4,19]. However, the transient influx of cytoplasmic proteins into the nucleus (e.g. YAP) as well as the accumulation of DNA to cyclic GMP-AMP synthase (cGAS) factor at the cytosolic side are processes that can be ascribable also to Lamin A/C structural ruptures due to the effects of cell migration into strict geometrical constrictions or cytoskeletal tensions acting on the nucleus [20,21]. Thus, both compressive and tensile forces can cause ruptures of the Lamin A/C, inducing an enhanced exposure of the nuclear content to the cytoplasm and possible DNA damages [22,23]. In fact, diffusion experiments of macromolecules into the cell nucleus reveal that their movement can be affected by viscosity, active transport, or the presence of obstacles such as the Lamin A/C structure and chromatin condensation, as well as by their dimension and molecular weight [24,25]. This implies that the application of an external force on the cell nucleus and its consequent deformation could favour molecule trafficking across the NE. In quasi-suspended cells, regardless of the cytoskeleton formation, it was demonstrated that forces directly applied up to the nucleus level contribute to a nuclear YAP translocation, by decreasing the NE mechanical resistance [8]. In fact, nuclear flattening can cause a NPCs stretching which leads to an enhanced YAP shuttling as well as the passage of external molecules into the nucleus [8]. Until now, an in-depth study of calibration of external forces on suspended cells suitable to induce mechanical nuclear responses by changing the NE structural conformation and/or triggering shuttling pathways has been missing. In recent years, there has been a substantial growing interest in measurement techniques to deform cell nuclei with controllable NE modifications (e.g. localized Lamin A/C damages) by also inducing rapid delivery of molecules into the nucleus. Therefore, microfluidic-based approaches emerged as high throughput techniques with highly controllable characteristics useful for nucleus stimulation and efficient intracellular biomolecule delivery [18]. For instance, an electric field can be used to induce a transient NE disruption, which favours the entry of a target material before a resealing mechanism starts [18]. However, the external application of strong electric fields could reduce cell viability, encouraging a more accurate calibration of the field strength. Some other microfluidic approaches, mimicking micropipette aspiration technique, measure cellular ability to deform their own nuclei into microfluidic constrictions smaller than the size of the cell itself [15,24]. Experimental evidence shows that Lamin A/C levels highly affect the ability of cells to pass through microfluidic constrictions, whereas the nucleus shape appears to be less important [15,26]. Major drawbacks of these microfluidic approaches are cell clogging and low throughput. Instead, simple fluid-flow shear forces can deform cells, even though, in this case, a proper calibration and variation of the hydrodynamic force is necessary [27–29]. On the other hand, in-flow tuneable viscoelastic compressive forces offer the unique possibility to induce controlled and variable levels of deformation on cells, for large sample numbers and in a completely contactless and viable way [30]. By using in-flow tuneable viscoelastic compressive forces to determine different levels of cell deformation, this microfluidic approach already provided a mechanical phenotyping of breast cell lines by detecting in-flow deformation-dependent dynamics. Here, we show how precise control over such in-flow compressive forces induce specific levels of cell nucleus deformation, consequently regulating NE alterations in terms of Lamin A/C ruptures or expression changes as well as chromatin re-organization phenomena. According to this approach, suspended single cells are forced to pass into a microfluidic rectangular cross-section, where viscoelastic fluid forces compress cells [30]. The flowing fluid is a biocompatible polymeric viscoelastic solution (polyethylene oxide, PEO), highly adaptable in rheological properties in order to easily change the applied compression level—ranging from 10 µN to 600 µN—by changing polymer concentration and flow-conditions as a function of cell size. We demonstrated that these tuneable compressive forces reach the nucleus structure inducing cell-specific nuclear deformation and, consequently, Lamin A/C modifications of different entities as occurs when cells are cultured on substrates of different stiffness. A resulting enhanced NE permeability led us to explore the correlation between Lamin A/C modifications and altered phenomena of molecule influx–efflux, verifying how the entry of Hoechst and YAP molecules can be influenced by Lamin A/C changes. Therefore, our results show a more rapid re-localization of these molecules after increased compression. Lamin A/C ruptures correlate with a decreasing chromatin content and a cGAS activation after the highest compressive force. Our proposed microfluidic-based measurement technique allows to appreciate new insights in studying cell mechanobiology proposing a new way of mechano-diagnosis in cancer research. By wide-range of applied stress, our approach allows to induce a rapid nuclear cell response, depicting different degrees of Lamin A/C and chromatin alterations directly correlated to enhanced NE permeability and consequently to a re-localizing of intracellular molecules. Therefore, the used microfluidic device permits to cover a wide-range of applied stress on single cell, highlighting unprecedented nuclear response with reduced time and cost for more detailed nuclear mechanics investigations.

## 2. Results and discussion

### 2.1. Nuclear deformation upon different levels of in-flow compressive forces

**Figure 1.**
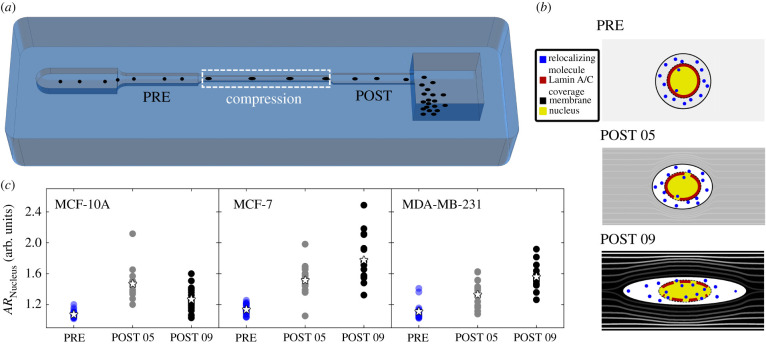
In-flow nucleus deformation analysis. (*a*) Cell nuclei were observed and analysed by in-flow fluorescent acquisition. The ARNucleus parameters are quantified at PRE and POST. (*b*) Nuclei are then analysed off-chip before (Control) and after (PEO 05 and PEO 09) the in-flow deformation. (c) ARNucleus parameters are reported for each cell line at the different in-flow measurement points at the PEO conditions. All cell lines start from the same mean value of ARNucleus (stars) measured at PRE (blue). Then, except for MCF-10A (PRE *n* = 29; POST 05 *n* = 12; POST 09 *n* = 27), both MCF-7 (PRE *n* = 27; POST 05 *n* = 20; POST 09 *n* = 18) and MDA-MB-231 (PRE *n* = 19; POST 05 *n* = 17; POST 09 *n* = 15) undergo higher nucleus deformation for increasing levels of compression. For statistical analysis, a Kruskal-Wallis test was performed. Significant differences between PRE and POST, both 05 and 09, were found for all three cell lines (*p* < 0.001).

To determine nuclear deformation and relative NE responses as function of different in-flow compressive forces, we defined both on-chip and off-chip measurement steps. We performed the on-chip analysis of nucleus deformation, defined as nuclear aspect ratio (*AR*_Nucleus_), at two different measurement positions (PRE and POST, figure 1*a*) applying a low or high compression level corresponding to two different viscoelastic PEO concentration, identified as 05 (low compression) and 09 (high compression). PRE investigation was used to confirm the absence of nucleus deformation before compression (figure 1*a*, white dashed box) since the applied force needs to align cells [30]. Instead, POST compression measurements were used to monitor possible nucleus deformation (figure 1*a*). More precisely, the compression was applied with a pressure-driven flow of the viscoelastic fluid into a microfluidic device, where cells deform under the action of fluid streamlines, in a completely contactless way (figure 1*a*) [30]. Compression forces on the whole cells were estimated to act in the range of (10–10^3^) µN (see electronic supplementary material, table S1), which enabled the study of different cell responses over a wide range of mechanical loading conditions. The force computation takes into account the rheological properties of the viscoelastic fluid, specified by the chosen flow condition, and the cell size and position in the microfluidic channel [30]. Accordingly, bigger cells perceive higher compression forces with respect to smaller ones. In fact, the viscoelastic concentration choice relies on the need to reach the cell nucleus and the relative constituents with forces of variable entity. Specifically, in our experiments, MCF-10A and MDA-MB-231 were found to be dimensionally similar, experiencing a comparable compression level corresponding to approximately 20 µN and approximately 200 µN at PEO 05 and PEO 09, respectively, whereas MCF-7 experience a higher force level of approximately 600 µN. We recently demonstrated that, at PEO 05 and PEO 09, MCF-10A and MCF-7 stiffen, whereas MDA-MB-231 tend to soften as the applied force increases [30]. Such force-dependent behaviour was associated with the nucleus, as significantly more rigid in MCF-10A and MCF-7 compared to MDA-MB-23 [31]. Thus, we decided to monitor the in-flow *AR*_Nucleus_ variation to estimate whether a nuclear deformation occurs at the two different compression conditions. As expected, where forces are not high enough (PRE) to compress the whole cells [30], nuclei do not deform (*AR*_Nucleus_ ∼ 1) in each cell line (figure 1*c*). Differently, immediately after the application of the compressive forces (5 s) in compression region, we measured increasing *AR*_Nucleus_ values, as PEO concentration becomes higher. Interestingly, MCF-10A express a higher deformation at PEO 05 than at PEO 09. This response is ascribable to a mechanical stiffening of the nucleus structure at the highest compression. On the contrary, MCF-7 and MDA-MB-231 gradually deform their own nucleus, reaching up to 50% and 40% increase of *AR*_Nucleus_, respectively (figure 1*c*).

To demonstrate the versatility of the presented approach on cells that are grown in suspension, we tested a smaller Jurkat human leukaemia T cell line (approx. 10 µm-diameter) at both compression levels. Since the compression level is inversely related to the cell size, we found that only the highest compression was able to induce a significant nuclear deformation (∼30% increase of *AR*_Nucleus_ from PRE to POST 09, see electronic supplementary material, S1).

To evaluate the impact of the applied compression on nuclear structure, an off-chip cell nucleus analysis was performed. We defined a quiescent pre-deformation condition (Control) and two after-in-flow conditions as PEO 05 and PEO 09, respectively. In particular, we conducted image analysis of Lamin A/C structure and chromatin condensation state after in-flow compression, correlating their modifications to re-localization phenomena of molecules (e.g. YAP and Hoechst; figure 1*b*). To perform a proper image acquisition, cells were allowed to slightly adhere. To prevent protein level alterations due to degradation, recycling or production processes ascribable to the cell adhesion—and not to the imposed compression—an adhesion time of 10 min was chosen [17].

### 2.2. Lamin A/C content scales by applied in-flow tuneable compression

**Figure 2.**
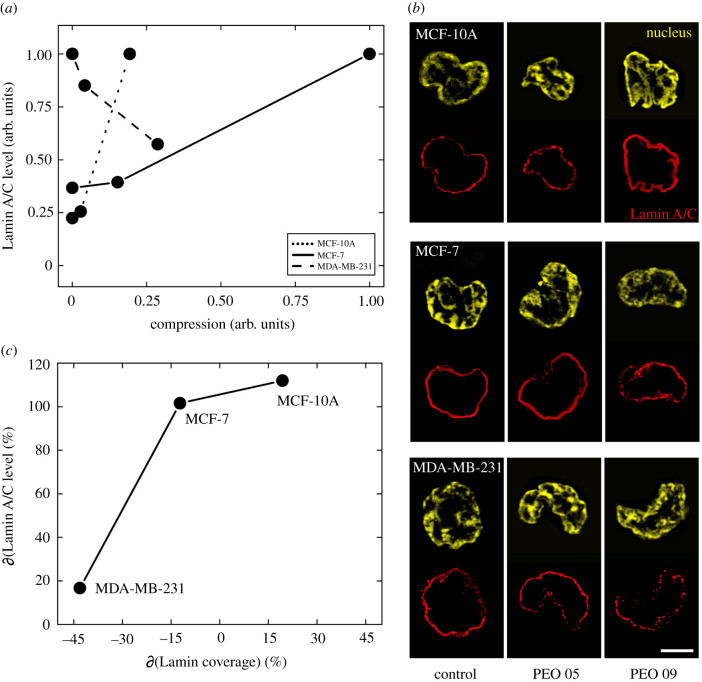
Cell-dependent Lamin A/C changes after in-flow compressive forces. (a) A correlation between the applied compression and the Lamin A/C levels for each cell line is presented. MCF-10A were found to be more sensitive to the applied compression than MCF-7, since the Lamin A/C protein level appears to be increased at PEO 09. On the contrary, MDA-MB-231 show a decreasing trend of the protein level as the force enhances. (*b*) The variation values of the Lamin A/C level and coverage are reported in the case of PEO 09 compared to the Control condition for each cell line. As expected, increasing values of Lamin A/C level correspond to a greater coverage in MCF-10A, as well as decreasing protein levels coupled with a coverage disruption in MDA-MB-231. An interesting finding is that MCF-7 enhanced the Lamin A/C level but decreased in coverage content, possibly due to protein production phenomena. (*c*) Confocal images of Lamin A/C and nucleus of the three cell lines tested in the three different experimental conditions are reported, highlighting the behaviour of increased coverage in MCF-10A (*n* = 20, *n* = 25, *n* = 17 for Control, PEO 05 and PEO 09, respectively) and disruption in MCF-7 (*n* = 25, *n* = 26, *n* = 23 for Control, PEO 05 and PEO 09, respectively) and MDA-MB-231 (*n* = 26, *n* = 19, *n* = 16 for Control, PEO 05 and PEO 09, respectively), coupled with the corresponding level variations. Images are of the middle z-section of different nuclei. Reported scale bar is 5 µm. For statistical analysis see electronic supplementary material S2 and S3.

As Lamin A/C is one of the major compartments involved in mechanical cell nucleus deformation, we investigated the relative responses to the different in-flow forces. Lamin A/C level was expressed as normalized value of signal intensity on the maximum value reached by each cell line upon the different compression degrees. The latter are defined as normalized values of forces with respect to the maximum applicable force into the microfluidic device, evaluated on the biggest size of cell tested. Lamin A/C coverage represents another relevant parameter. It was measured locating the constituents parts of the structure with respect to the nuclear perimeter of cells. From the comparison of these two entities, we extract the Lamin A/C amount covering the nucleus. At Control, cell lines show different initial Lamin A/C expressions. Of interest, the increase of the Lamin A/C with the compression is more pronounced in MCF-10A than in MCF-7 even though the entity of the perceived fluid force is higher for MCF-7, resulting in the maximum compression level (equal to 1, figure 2*a*). Thus, our results suggest that MCF-10A are more sensitive to the imposed compression (figure 2*a,c*). On the other hand, MDA-MB-231 show a decreasing Lamin A/C content in terms of signal intensity. We noticed an enhanced expression level and coverage of Lamin A/C, for MCF-10A, possibly due to a recycling or a new production of the Lamin A/C protein associated with the highest in-flow applied compression.

As expected, MCF-10A were characterized by typical wrinkles due to the induced non-adherent condition (figure 2*c*) [32]. On the contrary, with respect to the Control, MCF-7 slightly decreased in Lamin A/C coverage, despite the increase in the level amount. This response might be due to the necessity for the cell to produce new protein in order to activate a repair process of the impressed damage. A destroyed Lamin A/C is evident in the case of MDA-MB-231 that gradually deconstruct the structure as the force increases, leading to disruption (figure 2*b,c*). In cancer cells, especially breast cells, it is well known that lower levels of Lamin A/C correlate with a higher degree of metastatic potential [14,22]. Thus, nuclei lacking Lamin A/C deformed more easily, allowing the invasion of surrounding tissues. However, a removal of Lamin A/C could lead to an increased cell death and then reduced metastasis. It has been demonstrated that under elevated shear stress conditions, MDA-MB-231 resulted to be more resistant to a shear force-induced apoptosis than MCF-10A, despite having a more compact and less deformable Lamin A/C than MDA-MB-231 [26,31] We hypothesize that, under in-flow compression, the cellular response changes by reversing the trend, with MCF-10A more resistant than MDA-MB-231 at the Lamin A/C level. Following the same principle, we tested cell vitality by observing how the recovered cells after the in-flow compression adhere on a plastic substrate (approx. 2 GPa) (see electronic supplementary material S4). For each line and applied force cells spread on the substrate, after at least 3 h of adhesion, except for MDA-MB-231 at the highest compression. This suggests functional cell activities are not fully recovered, mainly due to a not reversible Lamin A/C damage that triggers a cell death process. Previous studies demonstrated that in MDA-MB-231 a suspension state increased the adhesion and the cytoskeleton formation because of Lamin A/C upregulation [33]. These findings suggest that, despite their suspension state, our applied in-flow compression inhibits MDA-MB-231 adhesion after Lamin A/C rupture and reduced level expression. On the contrary, MCF-7 respond differently after the in-flow compression by recovering their own functional vitality despite the observed Lamin A/C damages.

### 2.3. Lamin A/C modifications correlate with YAP shuttling phenomena

**Figure 3.**
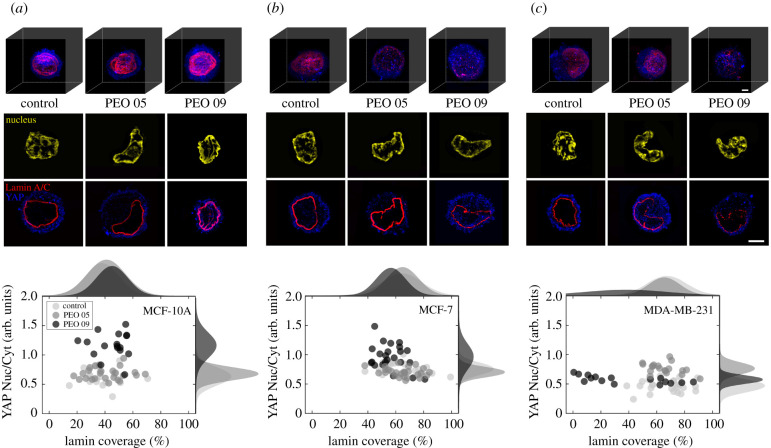
YAP shuttling phenomena associated to Lamin A/C changes. (*a*) On the top, representative three-dimensional fluorescent images of MCF-10A expressing green Lamin A/C and red YAP at the three different measurement conditions, performed with a confocal microscope (Three dimensional reconstruction with ImageJ Plugin Stacks – Z-functions, 360->3D Project Macro). In the middle, single confocal z-stack images of Lamin A/C and YAP tested in the three different experimental conditions are reported, highlighting the behaviour of increased coverage and gradual YAP nuclear content increment up to PEO 09. On the bottom, a scatter plot representation of the YAP Nuc/Cyt-ratio with respect to Lamin A/C coverage is reported describing how no relevant changes are expected at PEO 05 but only in the higher values of the parameters at PEO 09 with respect to the Control condition (*n* = 20, *n* = 25, *n* = 17 for Control, PEO 05 and PEO 09, respectively). (*b*) On the top, representative three-dimensional fluorescent images of MCF-7 in the three analysis conditions. In the middle, confocal images of Lamin A/C and YAP highlight that Lamin A/C slightly decreases in coverage while YAP nuclear content enhances at PEO 09. On the bottom, a scatter plot representation of the YAP Nuc/Cyt-ratio with respect to Lamin A/C coverage confirms how a competition between a mechanosensitive process and an increasing of the YAP nuclear signal due to an enhanced nucleus permeability is possible, at PEO 09 condition (*n* = 25, *n* = 26, *n* = 23 for Control, PEO 05 and PEO 09, respectively). (*c*) On the top, representative three-dimensional fluorescent images of MDA-MB-231, clearly defining the gradual disruption of the Lamin A/C structure. In the middle, confocal images show that Lamin A/C coverage decreases as well as the YAP signal, at the highest PEO concentration. Differently, at PEO 05, a competitive mechanism for the increasing YAP Nuc/Cyt-ratio, as described for MCF-7, is appreciable. Such outcome are confirmed by the scatter plot representation, reported on the bottom (*n* = 26, *n* = 19, *n* = 16 for Control, PEO 05 and PEO 09, respectively). Images are of the middle z-section of different nuclei. Reported scale bar is 5 µm. For statistical analysis see electronic supplementary material S5.

In recent years, it has been demonstrated that a direct force application on the nucleus leads to an increased YAP influx into the nucleus [19]. To study whether our in-flow compression generates similar cell responses, we investigated YAP nuclear signal. In Control, all cell lines express a well-defined YAP cytoplasmic signal (figure 3*ac*, top row). Both MCF-10A and MCF-7, at PEO 05, show not altered Nuc/Cyt ratio and Lamin A/C coverage. Scatter plots highlight cell-specific trends of response (figure 3*ac*, top row). In fact, MCF-10A at PEO 09 show an evident increase of the YAP Nuc/Cyt-ratio as well as the Lamin A/C content (figure 3*a*). In particular, the shuttling of mechanosensitive YAP suggests that thickening and an enhancement of the Lamin A/C content regulate the nuclear expression of the transcription factor, with a two-fold increase of YAP signal after the in-flow compression. It is known that, on stiff substrates, cells with high expression levels of Lamin A/C present a higher nuclear YAP content, suggesting that the Lamin A/C changes influence nuclear localization of transcription factors [19,32]. Thus, similar effects are appreciable by inducing a cell deformation up to the nucleus level in our conditions. MCF-7 show a different scenario. The YAP Nuc/Cyt-ratio increases, defining a shuttling phenomenon due to a double effect of mechano-regulated response and enhanced nuclear permeability at the ruptures' localization of the Lamin A/C. In fact, scatter plots show a shifting at lower levels of coverage corresponding to higher values of Nuc/Cyt-ratio, suggesting that some points of damage are present at the Lamin A/C level favouriting the molecule re-localization (figure 3*b*, bottom row). However, previous studies described YAP protein levels as decreasing with Lamin A/C knockdown and then with the relative NE losses [17,32]. MDA-MB-231 outcome show decreasing or unaltered values of YAP Nuc/Cyt-ratio at PEO 09, with Lamin A/C deconstruction. Therefore, the MDA-MB-231 inability to mechanosense the applied compression and to perform a YAP shuttling phenomenon might be ascribable to a loss in cell vitality. Conversely, at PEO 05, MDA-MB-231 behave comparably to MFC-7, by slightly decreasing in Lamin A/C coverage but with a small increase in YAP Nuc/Cyt-ratio (figure 3*c*, on the bottom). Further, the mentioned phenomena might be attributed to a variation of the mechanical stability and/or molecular weight of YAP if bound/unbound to other molecules [19].

### 2.4. Lamin A/C modifications affect cell nuclear permeability and chromatin condensation states, revealing cGAS cytoplasmic activity

To test whether, in our conditions, the reduction of Lamin A/C coverage promotes enhanced nuclear permeability and chromatin condensation changes, we measured and analysed the nuclear entry of the Hoechst 33 342 molecules. After having recovered cell samples subjected to in-flow compression, they were directly fixed to evaluate the kinetics of Hoechst from the cytoplasm to the nucleus. In greater detail, after loading the Hoechst solution in contact with the cell sample, we monitored the kinetics of molecule entry for 10 min, to reduce undesired cell movements, saturation or bleaching signal events [34].

**Figure 4.**
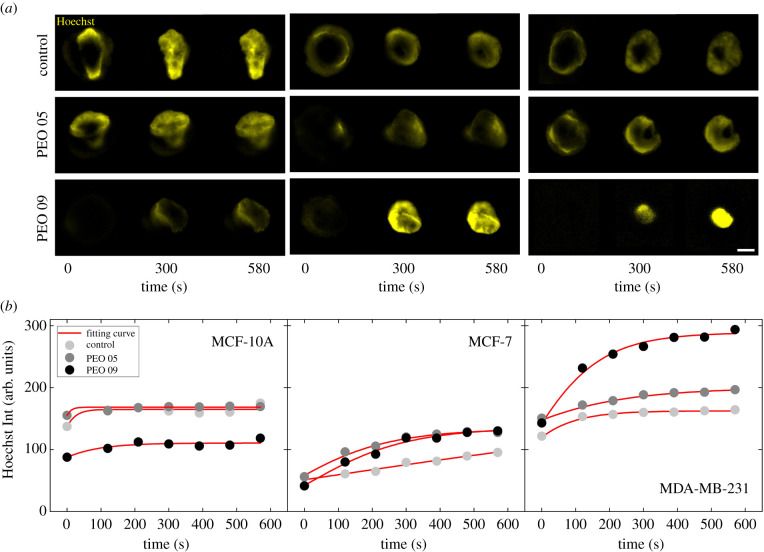
Recording Hoechst molecule entry into the cell nucleus reveals an enhanced nuclear permeability after in-flow compression. (*a*) Microscope images of cell nuclei stained with Hoechst 33342 solution at three time points (0, 300, 580s) of the entire kinetics of Hoechst entry for a total period of 10 minutes. On the left, MCF-10A show that, at PEO 09, Hoechst nuclear delivery from the cytoplasm to the nucleus is hindered by the thicker Lamin A/C. In the middle, MCF-7 are represented. Both at PEO 05 and PEO 09 the nuclear influx is enhanced by the Lamin A/C changes, with a difference in the final reached intensity level that appears to be higher in the case of PEO 09. On the right, MDA-MB-231 gradually increase in nuclear Hoechst influx, reaching maximum values of both entrance velocity and final intensity level at PEO 09. (*b*) Fitting curves by application of Gompertz model are shown. The fitting procedure was performed on equally distributed time steps (*n* = 20) from 0 to 600 s. For sake of simplicity, we report the fitting curves with 7 relevant time points. In MCF-10A, at PEO 09 (*n* = 5, *n* = 4 and *n* = 5 at Control, PEO 05 and PEO 09, respectively), Hoechst intensity level clearly reduces in comparison with the Control condition, whereas MCF-7 (*n* = 7, *n* = 5 and *n* = 5 at Control, PEO 05 and PEO 09, respectively) and MDA-MB-231(*n* = 7, *n* = 4 and *n* = 7 at Control, PEO 05 and PEO 09, respectively) speed up the entry reaching higher levels of final intensity both at PEO 05 and PEO 09. MDA-MB-231 reach a twofold higher intensity value at PEO 09 with respect to Control. Reported scale bar is 5 µm. For statistical analysis see electronic supplementary material S6.

At Control, the Hoechst entry kinetics are cell-line dependent, resulting in different velocity and intensity levels reached during the phenomenon (figure 4*a*, Control). In particular, MCF-10A do not show relevant differences between Control and PEO 05, since both the intensity and the entry behaviour remain unaltered after compression (figure 4*b*, MCF-10A; see electronic supplementary material, figure S6, table S3, and movies S1 and S2). At PEO 09 we observe a slower passage of the Hoechst into the nucleus, due to the previously described Lamin A/C thickening, leading to a drastic reduction of the final nucleus intensity. MCF-7, instead, show an enhanced Hoechst mobility, particularly at PEO 09, where localized ruptures of the Lamin A/C allow for a facilitated passage of external molecules. However, similar variations on the final Hoechst nuclear intensity are present at PEO 05 and PEO 09 conditions (figure 4*b*, MCF-7; see electronic supplementary material, figure S6, table S3, and movies S3 and S4). The Hoechst kinetics results to be completely altered by the in-flow compression for MDA-MB-231. We observe a gradual increase of the Hoechst signal, clearly enabled by the Lamin A/C deconstruction which already starts at PEO 05 compression. A facilitated movement of the molecule is then possible thanks to an increased permeability. However, an almost saturated signal is observed at the last time point of the experiment at PEO 09 (figure 4*b*, MDA-MB-231; see electronic supplementary material figure S6, table S3, and movies S5 and S6).

**Figure 5.**
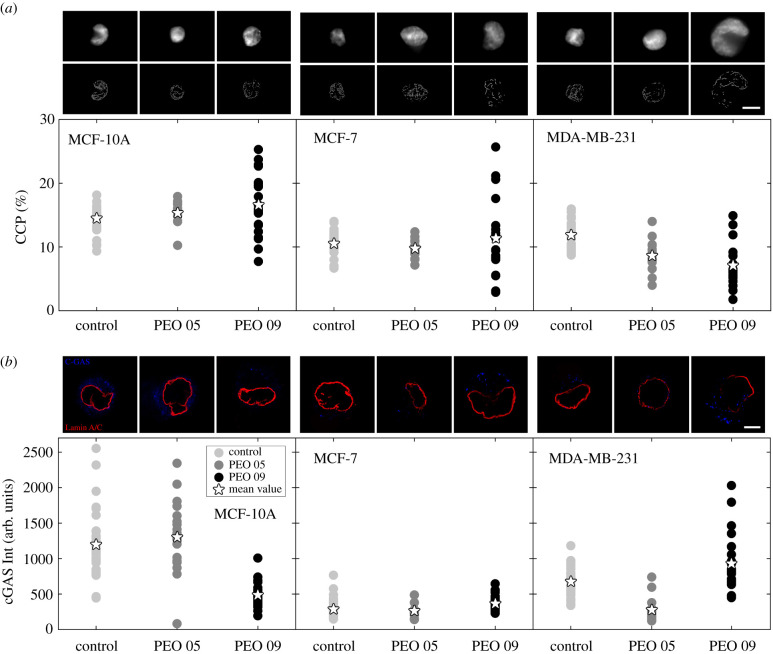
Chromatin condensation changes and cGAS localization on DNA damages are correlated with Lamin A/C ruptures. (*a*) Chromatin microscope fluorescent images and scatter data of CCP are presented for each cell line at the three different measurement conditions. MCF-10A and MCF-7 do not show relevant CCP changes, while MDA-MB-231 gradually decrease in chromatin condensation by increasing the applied compression (MCF-10A: *n* = 31, *n* = 10, *n* = 25 at Control, PEO 05 and PEO 09 respectively; MCF-7: *n* = 20, *n* = 13 and *n* = 20 at Control, PEO 05 and PEO 09, respectively; MDA-MB-231: *n* = 26, *n* = 18 and *n* = 20 at Control, PEO 05 and PEO 09, respectively). (*b*) cGAS intensity is reported for each cell line in the three different measurement conditions, as probing tool to verify whether a chromatin de-condensation and the DNA damages occur once the Lamin A/C ruptures. On the top, cGAS confocal images show a scattered initial signal into the cell cytoplasm without specific localization at DNA spots. This results to be evident at PEO 09 in MCF-7 and MDA-MB-231, whereas cGAS signal content decreases in MCF-10A at PEO 09 and MDA-MB-231 at PEO 05. On the bottom, scatter data plots confirm the mentioned trends of modification (MCF-10A: *n* = 34, *n* = 21, *n* = 30 at Control, PEO 05 and PEO 09 respectively; MCF-7: *n* = 39, *n* = 10 and *n* = 31 at Control, PEO 05 and PEO 09, respectively; MDA-MB-231: *n* = 32, *n* = 18 and *n* = 25 at Control, PEO 05 and PEO 09, respectively). Reported scale bar is 5 µm. For statistical analysis see electronic supplementary material S7 and S8.

Within the nucleus, Lamin A/C regulates DNA replication and repair as well as chromatin organization. In particular, heterochromatin exists at the nuclear periphery and interacts with the nuclear Lamin A/C at specific sites. These interactions may directly affect chromatin organization, nucleus mechanosensitivity and transcriptional activity [6,35–37]. For this reason, observing the changes of Lamin A/C at the various degrees of applied in-flow compression, we asked whether these modifications also translate at the level of the nucleus with alterations of chromatin condensation. Lamin A/C thickening does not confer a higher chromatin condensation to the nucleus in MCF-10A (figure 5*a*, top row). Scatter plots and relative mean values of chromatin condensation parameter (CCP) show not relevant modifications at the chromatin level are present at the two in-flow compression conditions (figure 5*a*, bottom row; see electronic supplementary material, figure S7). The situation is similar for MCF-7, despite a different chromatin content already at the Control condition (figure 5*a*, on the bottom, see electronic supplementary material, figure S7). A decreasing trend of chromatin condensation is observed in MDA-MB-231, which can be associated with the previous Hoechst molecule outcome. In fact, chromatin density and condensation could affect nuclear influxes, hindering or not the passage of molecules [24]. However, Lamin A/C ruptures coupled with chromatin losses reveal possible reasons for the MDA-MB-231 failure in vitality. Scatter plots clearly indicate the highest decrease of chromatin content at PEO 09 condition. To test whether chromatin losses coupled with DNA exit into the cytoplasmic region, we evaluated the activation of cGAS protein, which is a fundamental cytosolic DNA-sensor [38]. At Control condition, cell lines express a cGAS quote into the cytoplasm (figure 5*b*, top row) because not only cGAS is localized in the cytoplasm of nondividing cells, but it also associates to DNA foci once an accumulation of damaged DNA into the cytoplasm occurs [39]. However, scattered signals of cGAS without specific localization with DNA spots are present in our Control condition (figure 5*b*, top row). We observed that not relevant modifications in the scatter data and mean values of cGAS intensity are present in MCF-10A and MCF-7 after in-flow compression at PEO 05 (figure 5*b*, bottom row; see electronic supplementary material, figure S8). Notably, at PEO 09, MCF-10A show a decreasing level of cGAS intensity, which suggests that a possible protein content modification has occurred. A similar result was observed at PEO 05 in MDA-MB-231. At PEO 09, well-defined spots of active cGAS attached on damaged DNA are present in MCF-7 and MDA-MB-231. The latter shows that such cGAS content colocalizes or results to be close to the Lamin A/C ruptures. cGAS further confirmed that Lamin A/C damage occurred at the highest compression conditions opening the nucleus to the cytoplasmic side and relative induced pathways. Remarkably, the substantial enhancement of cytosolic cGAS expression may restrain DNA repair and evoke cell death, as observed in MDA-MB-231 at PEO 09 condition [40].

## 3. Conclusion

By exploitation of in-flow viscoelastic tuneable compression forces, the presented microfluidic approach is a suitable tool to rapidly compress cells up to the nucleus level in a highly controllable manner, unveiling the possibility to activate pathways and responses generally ascribable to adhesive and migratory cell behaviour. Our microfluidic approach with tuneable fluid-flow conditions offers the possibility to deform the cell nucleus in a completely contactless and viable way—with a wide-range of compression forces from *μ*N to mN—eliciting cell-specific responses, in terms of protein modifications as Lamin A/C production or recycling, structural thickening or deconstruction. Moreover, at the highest applied compression, we present a chromatin condensation change, reached with a relative DNA alteration and damage. The mentioned responses stimulate processes that have usually been observed with external stimuli coming from variable substrate rigidities, different migration geometries, compression micropipette conditions or in-flow shear forces. Our compressive forces result to be quantitatively equal to forces that would be obtained in adhesion conditions, with well-structured cytoskeletal components in response to substrate rigidity, or to stimuli that would come from confinement configurations for migration. Indeed, we show that after the applied flow, MCF-10A respond with a thicker Lamin A/C structure and a higher protein level coupled with an enhanced nuclear YAP signal, suggesting that well-known mechanosensing cell responses could occur regardless of the presence of a defined cytoskeleton structure. Moreover, we observe that cell-dependent responses are obtained as a function of applied compression forces, demonstrating that the mentioned mechanobiological responses are not only tissue-specific but also cell-line-related. In addition, the thicker Lamin A/C slows down the passage of a probing molecule like Hoechst, reducing the entry time as well as final intensity level into the nucleus. However, such slower passage of Hoechst molecules could be addressed also to an inner chromatin re-organization in the case of MCF-10A, showing an increasing trend of CCP values. Furthermore, the decrease of cGAS expression indicates the ability of healthy cells to activate structural modifications of the nuclear components, after the application of the maximum compression level, avoiding DNA misplacement. On the contrary, at PEO 09 (approx. 600 µN), MCF-7 highlights recovering phenomena of the Lamin A/C structure that shows reduced coverage but enhanced protein level, defining possible conditions for new protein production to restore the effects of the applied compression. The slightly appreciable cGAS activation supports the hypothesis that cell recovery occurs after the imposed load condition. On the other hand, YAP nuclear level increases, with combined mechanisms of mechanosensing responses and enhanced NE permeability after Lamin A/C deconstruction. Similarly, at PEO 05 (approx. 20 µN), MDA-MB-231 express such a competing mechanism for YAP nuclear influx, leading to slightly higher levels of YAP nuclear content. Further, Hoechst molecule entry across the NE is facilitated and accelerated, both for MCF-7 and MDA-MB-231 at PEO 09 and PEO 05, respectively. The highly invasive MDA-MB-231 shows a gradual Lamin A/C modification, up to a definite level of disruption at PEO 09 (approx. 100 µN), which induces a loss in cell functionality and a consequent increased cell mortality. Thus, an interplay between the loss of Lamin A/C structure integrity and cell vitality could be recognized, assigning to the Lamin A/C a central role in the induced cell mortality, clearly associated also to the exposure of a decondensed chromatin and the relative cell DNA damage. In this respect, our approach provides a cell-specific threshold value of compressive forces for cell vitality. In conclusion, in-flow tuneable viscoelastic compressive forces offer the possibility to calibrate nucleus mechanical responses, triggering mechanosensing reactions or an enhanced nuclear permeability as well as chromatin re-organization and losses in cell functionalities. For future investigations, our approach appears to be interestingly applicable also for promoting enhanced opening of NPCs, leading to similar effects of nucleus stretching by playing with fluid-flow conditions and compressive force entities. Then, it is of experimental interest whether the use of our microfluidic approach will be able to create a complete mapping of nuclear mechanics responses and relative underlying mechanisms, to elicit different levels of cell nucleus reaction depending on the chosen compression condition. Moreover, our approach highlights great potential in investigating the mechanical nuclear responses of different cell types, such as circulating tumour cells or immune system cells, for which enhanced nuclear permeability evidence and a precise cell mechanosensing analysis have gained increasing attention.

## 4. Material and methods

### 4.1. Viscoelastic compressive forces

To induce deformation of single cells under viscoelastic flow conditions, we used a highly biocompatible PEO (4MDa, Sigma Aldrich). Fluid rheological properties have been previously investigated and properly characterized [30]. We decided to work with two different PEO concentrations, in order to induce two distinct levels of in-flow compressive forces. In particular, we selected PEO 0.5 and 0.9 concentrations, which correspond to polymer concentrations of 0.53 and 0.88, respectively. We calibrated the initial pressure and velocity conditions into the microfluidic device, as well as the channel geometry in order to align and deform cells in a contactless way. The same fluid-flow velocity was imposed for both PEO 05 and PEO 09, in order to establish a comparable flow condition. We computed viscoelastic compressive force (*F_EMax_*) values coming from the channel walls (see electronic supplementary material, table S1), supposing the cell already at its own equilibrium position where the competing viscoelastic (F_E_) and drag (F_D_) forces are balanced [30].

### 4.2. Experimental setup for the in-flow measurement

The setup includes a pressure pump (P-pump, Dolomite Microfluidics), a round shaped flexible fused silica capillary tubing (Molex) an ad-hoc designed microfluidic chip, a fluid connector (N-333, IDEX) and an inverted microscope (X81, Olympus) with CMOS camera (ORCA FLASH 4.0, Hamamatsu Photonics K.K.). Briefly, a pressure pump pushes the sample volume through the capillary in the microfluidic device inlet, while at the end of the chip a reservoir collects cells. The chip is made of two separate parts of poly methyl methacrylate, which are placed together by magnetic forces. In detail, we integrated one array of magnetic cubes (5 × 5 mm) in the main part and coincided another array of magnets on the cover part of the chip, capable of opening the whole chip. The main part was machined using a standard CNC based micromilling technique (Minitech Machinery) to develop microfluidic duct sections of different height, width and length (first, 250 × 500 × 10 000; second, 70 × 200 × 4000; third, 25 × 100 × 40 000; fourth, 90 × 200 × 10 000 µm, respectively), while the cover part was simply used to close the chip.31 In particular, a capillary is used to perfectly align cells in flow. Furthermore, the first two microfluidic sections (PRE) are designed to observe aligned cells without having relevant deformation effects. The subsequent section (Compression) drastically reduces the height of the duct (25 µm) and implies a well-defined viscoelastic force over a certain time period on each passing cell, enabling precise cell deformation in flow. Of note, the microfluidic chip is designed for tuneable applied compression forces in gradient direction (top and bottom), with neglectable deformation forces from the side walls. However, a subsequent enlarged section (POST), reduces the fluid velocity before collecting cells into the reservoir.

### 4.3. Cell culture

We investigated MCF-10A, MCF-7 and MDA-MB-231 cells. MCF-10A were donated by Prof. S. Piccolo (Istituto FIRC di oncologia molecolare, IFOM, Milan, Italy) and cultured in mammary epithelial basal medium (MEBM) supplemented with the mammary epithelial growth media (MEGM) bullet kit (Lonza). MCF-7 and MDA-MB-231 cell lines were kindly donated by Daidone's group and Dr. P. F. Cammarata (Institute of Molecular Bioimaging and Physiology, IBFM-CNR, Cefalù (PA), Italy), respectively. MFC-7 cells are cultured in eagle's minimum essential medium (EMEM, Sigma-Aldrich) containing 10% fetal bovine serum (FBS), 100 µg ml^−1^ l-glutamine and 100 U ml^−1^ penicillin/streptomycin. MDA-MB-231 cells are cultured in a 1 : 1 mixture of Dulbecco's modified essential medium (DMEM, Euroclone) and Ham's F-12 medium (Microtech) supplemented with 10% FBS, 1% non-essential amino acid mixture and 100 U ml^−1^ penicillin/streptomycin.

Finally, each investigated cell type was diluted in 500 µl of viscoelastic medium to reach a final cell concentration of about 50 cells per µl. Furthermore, cells were checked for *Mycoplasma* infection using Hoechst 33 342 (Life Technologies) DNA staining. We did not observe the presence of stained dots outside the nuclei by using an inverted microscope (X81, Olympus) equipped with a water immersion objective (60× objective with NA 1.35), showing no evidence of *Mycoplasma* infection.

### 4.4. Measurement procedure

We defined different on-chip steps as PRE, POST 05 and POST 09, refereed to the microfluidic sections and the PEO conditions at which the nuclei were analysed. At PRE, measurements were performed only with PEO 09 to confirm that still at the highest compression level no nucleus deformation occurs in such section. Then, POST 05 and POST 09 represent the measurement steps relative to the deformed nucleus configuration analysis, immediately after the section where the contactless compression occurs (white dashed box in figure 1*a*). This experimental choice relies on the specific device configuration which accounts for an enlarged section optimized for deformed cell observation [30]. On the other hand, Control and PEO 05 or PEO 09 are designed to be the quiescent measurements of stained cells before and after in-flow compression at the two PEO conditions (off-chip). We decided to test cells in Control condition both with PEO 05 and 09 in order to appreciate possible differences in the not-deformed configuration depending on the chosen PEO solution. In order to do this, we added PEO 05 and PEO 09 diluting them with the medium where cells have been treated. The experimental procedure consists of two different positions. Firstly, we performed in-flow cell nucleus investigations. In a typical experiment duration of 20 s, approximately 0.74 µl of cell suspension is pushed through the chip and investigated by the imaging system (approx. 50 cell µl^−1^), resulting in a total amount of approximately 35/measurement (cell to cell distance of more than 200 µm in Compression). Of note, experiment duration was limited to guarantee constant acquisition rate of 500 frames per second. However, to enable a versatile tracing of morphometric cell nucleus in flow condition, we use a 20× objective and a field of view of 2048 × 200 squared pixels (0.325 µm px^−1^), which covers a final cell tracing length of 1.33 mm in the beginning of POST. Of note—for all experiments—we performed nucleus investigations with the same velocities and time of force application (approx. 5 s) in Compression for each cell line. Secondly, cells are collected in the chip reservoir for 10 min (approx. 40 µl of sample volume) for further off-chip investigations. We performed Lamin A/C and nucleus investigations at Control, PEO 05 and PEO 09 conditions using a confocal microscope (TCS STED CW, Leica) in a modality resonant scanner (8 Hz), equipped with a 100× oil-immersion objective (NA 1.4). Observing the Lamin A/C modification, we acquired images coupling YAP and Lamin A/C signals, again by using STED confocal microscope. Image resolution was fixed at 1024 × 1024 squared pixels with a 2× zoom factor. In addition, we collected three-dimensional representations of cells at Control, PEO 05 and PEO 09 conditions in MCF-10A and MDA-MB-231 as representative cases of the obtained results. Image stacks, covering the total cell volume, were collected at 0.27 µm Z-spacing using a 63× oil immersion objective (NA 1.4) of a confocal laser scanning microscope (LSM 710, Zeiss) equipped with an argon and HeNe laser lines at the wavelengths of 488 and 543 nm, respectively. Image resolution was fixed at 512 × 512 squared pixels with a 4× zoom factor. Except for image stack acquisition, also cGAS-Lamin A/C images were collected with the LSM confocal laser microscope, with the same acquisition specifications. Hoechst entry and chromatin images were saved by using Olympus Cell-R equipped with a 60× water-immersion objective (NA 1.3).

### 4.5. Cell fixation and staining

Cells subjected to different viscoelastic forces were recovered after flux—10 min after measurement start—from the chip reservoir and seeded into separate wells of a μ-Slide (Ibidi). Technical needs for the performed confocal observation forced us to take the cell sample from the device allowing cells to settle and slightly adhere onto µ-Slide glass surfaces for 10 min. Although the proceeding time lasts longer than deformation time, applying the same cell settling and adhesion procedure at Control and PEO 05 and PEO 09 conditions, the different results can be exclusively ascribed to the applied compression. Moreover, this time window has been established as the maximum possible to avoid protein level alterations due to degradation, recycling or production processes ascribable to the cell settling condition and not to the imposed compression [17]. For immunostaining, cells were fixed with 4% paraformaldehyde (Sigma-Aldrich) for 15 min at room temperature, then rinsed twice with PBS. Permeabilization—with 0.1% Tryton X-100 (Sigma-Aldrich) for 5 min—was performed only for Lamin A/C, YAP and cGAS investigation. Thus, Lamin A/C was stained with monoclonal Lamin A/C mouse antibody (SC-376248) overnight at 4 °C and with Alexa488 goat anti-mouse secondary antibody. To analyse the Nuc/Cyt ratio of YAP, cells were incubated with primary YAP1 polyclonal rabbit antibody (PA1–46189, ThermoFisher Scientific) overnight at 4 °C and with Alexa543 goat anti-rabbit secondary antibody. Nuclei were counterstained with TO-PRO-3 stain (T3605, ThermoFisher Scientific) at room temperature for 30 min. cGAS staining was performed with primary cGAS rabbit polyclonal antibody (HPA031700 Sigma-Aldrich) overnight at 4 °C. cGAS antibody was donated from T. Panciera (University of Padova, Padova, Italy). Finally, cells were washed with PBS for three more times. To monitor the Hoechst entry into the nucleus, on not yet stained samples, we added a solution diluted at 1 : 1000 of concentration, by recording the molecule entry in 600 s of measurement [34] on cell samples previously fixed with 4% paraformaldehyde (Sigma-Aldrich) for 15 min at room temperature and then rinsed twice with PBS. Chromatin images have been collected at the end of the transitory time of Hoechst entry so that waiting 30 min as usual time for staining procedures. For the highest viscoelastic solution concentration, also a standard ‘Trypan blue test’ was performed, to monitor the cell viability over time. In particular, despite the absence of a functional vitality response in MDA-MB-231, we observe that a 100% of cell viability was guaranteed.

### 4.6. Experimental data analysis

Image analysis was carried out with ImageJ and Fiji softwares. In-flow cell analysis was performed at PRE, POST 05 and POST 09. We extracted for each investigated cell line the nucleus aspect ratio (*AR*_Nucleus_) parameter to detect the relative deformation. Nucleus aspect ratio (*AR*_Nucleus_) was computed as follows:

$$AR_{\mathrm{Nucleus}}=\frac{d_{1\mathrm{Nucleus}}}{d_{2\mathrm{Nucleus}}},$$

where *d*_1Nucleus_ and *d*_2Nucleus_ are, respectively, the major and the minor axis of the ellipse best fitting the nucleus at the beginning of the cell tracing region (section D).

For the Lamin A/C level quantification, we normalized the value of the single-channel intensity with respect to the area delimited by the Lamin A/C itself. Then, we defined the Lamin A/C coverage as the portion of the nuclear perimeter occupied by the Lamin A/C with respect to the entire available nuclear perimeter. We performed the present analysis, by using the ‘Analyse particles’ Fiji plugin on images with applied threshold, in order to detect the Lamin A/C constituent parts and then summing the single-particle perimeter to get the total Lamin A/C perimeter. We applied the same threshold value as well as Gaussian filtering definition for the images in order to make them in proper comparison. YAP signal was estimated by computing the ratio between the normalized integrated densities of the nucleus and of the cytoplasm, before detecting the respective nucleus and cell areas. For cGAS analysis, the single-channel intensity values were collected after a Gaussian filter of the image and correlated to the respective Lamin A/C. Hoechst intensity was measured by collecting the value of the nucleus area normalizing the intensity values on it. This procedure for each instant of time reveals how the signal changes during the time. To quantify the level of chromatin condensation, we processed fluorescence microscope images by using an edge detection algorithm. In more detail, the condensation of chromatin increases the number of distinct spaces within the nucleus, which can be detected by a Sobel edge detection algorithm (pixel reduction factor = 2 and Sobel threshold = 0.02). Thus, the normalized measure of the density of edges within the nucleus to its cross-section area gives a measure of the level of chromatin condensation defined as CCP [41].

### 4.7. Statistical analysis

For simplicity of reading results, we adapted all of the off-chip measured parameters of the after-flow PEO 09 condition with correction factors relative to Control in PEO 05 (see electronic supplementary material). Being conscious that a statistical comparison of the resulting data, with the correction factors, is not possible, we reported original raw data into box chart plots correlating them with the respective error bars defined by the application of a Kruskal–Wallis statistical test.

## Data Availability

All data needed to evaluate the conclusions in the paper are present in the paper and/or the electronic supplementary material. The datasets generated in this study are available from the corresponding author upon reasonable request. The file format of the raw data is .TIFF or .LSM, which can be read with ImageJ.
